# Effects of the Abdominal Draw-In Maneuver with Manual Resistance on Lumbopelvic Muscle Activity and Lateral Pelvic Tilt During Side-Lying Hip Abduction

**DOI:** 10.3390/medicina62050927

**Published:** 2026-05-10

**Authors:** Dong-Woo Kim, Hyung-Soo Shin

**Affiliations:** Department of Physical Therapy, College of Health, Kyungwoon University, Gumi-si 39160, Republic of Korea; kdwkjh@ikw.ac.kr

**Keywords:** gluteus medius, quadratus lumborum, side-lying hip abduction, abdominal draw-in maneuver, manual resistance

## Abstract

*Background and Objectives*: This study aimed to investigate the effects of the abdominal draw-in maneuver (ADIM) and manual resistance (MR), applied independently and with simultaneous application during side-lying hip abduction (SHA). Muscle activation patterns were assessed using the electromyographic activity of the gluteus medius (GM), quadratus lumborum (QL), and internal oblique (IO), along with the GM/QL ratio, and lumbopelvic control was assessed using lateral pelvic tilt (LPT). It was hypothesized that the simultaneous application of ADIM and MR during SHA would influence muscle activation patterns and lumbopelvic control compared to each intervention alone. *Materials and Methods*: Healthy young male participants (*n* = 22) performed SHA under three conditions: ADIM alone, MR alone, and ADIM with MR. Surface electromyography was used to measure muscle activity of the GM, QL, and IO. The GM/QL activity ratio and LPT angle were also assessed to evaluate lumbopelvic control. Statistical analysis was performed using repeated-measures ANOVA (*p* < 0.05). However, the findings may have limited generalizability to other populations. *Results*: The ADIM with MR condition significantly increased IO activity (*p* < 0.05, *d_z_* = 1.07, 95% CI [54.65, 62.26]) and the GM/QL ratio (*p* < 0.05, *d_z_* = 0.87, 95% CI [1.42, 1.73]) while reducing QL activity (*p* < 0.05, *d_z_* = −1.04, 95% CI [48.52, 56.24]) and lateral pelvic tilt compared to the MR condition (*p* < 0.05, *d_z_* = −1.85, 95% CI [4.88, 6.06]). In addition, GM activity was significantly higher in the MR and ADIM with MR conditions than in the ADIM condition (*p* < 0.05, *η_p_*^2^ = 0.812). *Conclusions*: The simultaneous application of ADIM and MR during SHA may enhance IO activation and the GM/QL ratio while reducing compensatory LPT. However, these findings should be interpreted as acute experimental responses, and their clinical applicability remains to be determined.

## 1. Introduction

Gluteus medius (GM) serves as a key muscle in hip abduction and contributes significantly to lateral dynamic stability of the hip and pelvis, playing an essential role in functional movements of the lower extremity [1,2,3]. The GM is the largest of the hip abductor muscles, representing roughly 60% of their total cross-sectional area [4]. During single-leg stance, the hip abductors are essential for maintaining pelvic stability, with the GM acting as the main stabilizing muscle [5,6]. Furthermore, weakness of the hip abductors has been associated with low back pain, and impaired function of the GM is closely related to various musculoskeletal disorders [7,8,9].

A commonly used method for strengthening the hip abductor muscles is side-lying hip abduction (SHA), which is performed in a gravity-resisted position with minimal weight-bearing demands [5,10]. SHA may facilitate preferential activation of the GM, making it particularly suitable for early-stage rehabilitation and targeted GM strengthening [5]. In addition, SHA provides a stable base of support, enabling controlled movement and facilitating preferential recruitment of the GM [11]. However, during SHA, compensatory movement of the quadratus lumborum (QL), a synergistic muscle, may occur instead of activation of the GM, which can hinder efficient muscle activation patterns [12,13,14,15,16,17]. Excessive activation of the QL has been associated with increased pelvic hiking and altered frontal plane pelvic mechanics, which may compromise lumbopelvic control and contribute to inefficient movement patterns [12,14,15]. Such compensatory patterns have also been linked to movement-related injuries and functional impairments, including low back pain, in previous studies [12,14,15]. Therefore, promoting preferential recruitment of the GM while reducing excessive activation of the QL is essential [13,16,17,18,19].

Therefore, strategies to minimize compensatory muscle activity by improving lumbopelvic control during SHA are necessary [13,18]. Lumbopelvic control facilitates efficient activation of target muscles by maintaining trunk and pelvic alignment and reducing unnecessary movements [16]. Insufficient trunk control may lead to increased compensatory activation of synergistic muscles such as the QL, thereby disrupting normal muscle coordination [19]. Accordingly, interventions that enhance lumbopelvic control are considered important for optimizing GM activation during SHA [13,18].

One of the most commonly used methods to enhance lumbopelvic stability is the abdominal draw-in maneuver (ADIM) [20,21]. ADIM selectively activates the transversus abdominis and internal oblique (IO), thereby increasing segmental stability of the spine and improving coordination among trunk muscles [22]. This mechanism helps suppress unnecessary activation of compensatory muscles and provides a more stable trunk environment during functional movements [23]. In particular, ADIM contributes to maintaining trunk stability during lower extremity movements, thereby facilitating efficient activation of the target muscles [13,20,21]. However, whether selective activation of the transversus abdominis directly leads to functional improvement remains controversial, and some studies have emphasized abdominal bracing, which promotes co-contraction of the trunk muscles, as an alternative approach [24,25].

Meanwhile, manual resistance (MR) is widely used as an effective method to facilitate preferential activation of target muscles [15,26]. By providing resistance directly, the therapist can precisely control the direction and magnitude of muscle contraction, thereby guiding the individual to perform more accurate movement patterns [20]. In addition, adjusting the direction and intensity of resistance can minimize compensatory movements and effectively increase muscle activation across various joint angles [27]. Due to these characteristics, MR is particularly useful as an intervention for neuromuscular re-education and preferential muscle activation, especially in the early stages of rehabilitation [20,27].

Previous studies on SHA have proposed various strategies to increase GM activation and reduce compensatory QL activity under different conditions [13,16]. Some studies have attempted to enhance GM activation and decrease excessive activity of the QL by applying trunk stabilization strategies or modifying body position [18,19]. In our previous study, the simultaneous application of ADIM and MR during prone hip extension demonstrated enhanced lumbopelvic control and increased activation of trunk and hip muscles [17]. However, these findings may not generalize to SHA due to differences in body position, loading, and muscle activation patterns. The combined application of ADIM and MR during SHA has not been sufficiently investigated, and it remains unclear whether this approach provides additional effects on muscle activation and lateral pelvic tilt (LPT) compared to each intervention alone. Therefore, the purpose of this study was to examine the effects of the simultaneous application of ADIM and MR during SHA on lumbopelvic muscle activity and LPT. In this study, lumbopelvic control was examined using EMG and LPT as observable indicators reflecting aspects of neuromuscular control. Through this approach, this study aims to explore a potential strategy to minimize compensatory movements and enhance preferential activation of the target muscles. It was hypothesized that the simultaneous application of ADIM and MR during SHA would result in altered lumbopelvic muscle activation patterns and LPT compared to ADIM or MR alone.

## 2. Materials and Methods

### 2.1. Participants

A randomized crossover design with a within-subject approach was adopted in this study, and all experimental procedures were completed in a single session. Data collection was carried out at Kyungwoon University (Gumi-si, Gyeongsangbuk-do, Republic of Korea) from January to March 2026. Twenty-two healthy adult male participants were recruited via university bulletin boards and announcements posted on the institutional learning management system (Table 1). Individuals who expressed interest in the study were asked to contact the research team and were enrolled after receiving a comprehensive explanation of the study objectives, procedures, and potential risks. Written informed consent was obtained from all participants prior to data collection. Ethical approval for this study was granted by the Institutional Review Board of Daegu University (IRB approval number: 1040621-202505-HR-039).

Healthy male adults in their twenties were recruited if they had no history of neurological, orthopedic, or functional disorders involving the lumbar spine, pelvis, or lower extremities, no structural deformities, and were free from current pain. Eligibility was confirmed through a self-reported health questionnaire and a physical examination conducted by the examiner to assess pain, neurological signs, and structural deformities. Individuals were excluded if they presented with current pain, previous surgery in the relevant regions, or any condition that could compromise safe performance of the SHA. Additionally, participants were excluded if they had participated in professional athletic training or regular resistance training within the past six months.

Based on previous studies with similar within-subject repeated-measures or crossover designs, the required sample size was estimated using G*Power 3.1.9.7 [20]. The sample size calculation was performed using an effect size f of 0.40, an alpha level of 0.05, and a statistical power of 0.95, indicating that at least 18 participants were required. In addition, a similar SHA study included 19 participants [19]. Considering possible dropouts, 22 participants were recruited in the present study.

The participants performed SHA under three conditions: (1) ADIM alone, (2) MR alone, and (3) ADIM with MR. To minimize potential carryover effects and muscle fatigue, a 5 min washout period was provided between each condition, which was considered sufficient based on previous studies involving similar isometric tasks. The sequence of the experimental conditions was generated using the RAND function in Microsoft Excel and assigned prior to data collection. Due to the nature of the within-subject crossover design, allocation concealment and blinding were not feasible. However, randomization and counterbalancing were applied to minimize potential order-related bias.

### 2.2. EMG Measurement and Signal Processing

Electromyographic data from the GM, QL, and IO during SHA were recorded using the TeleMyo DTS wireless surface EMG system (Noraxon Inc., Scottsdale, AZ, USA). Electrodes were placed at anatomically defined locations based on previously established protocols [13,18]. For the GM, electrodes were positioned midway between the greater trochanter and iliac crest. For the QL, electrodes were placed approximately 2–3 cm lateral to the L3 spinous process. For the IO, electrodes were positioned diagonally about 2 cm medial and inferior to the anterior superior iliac spine. Prior to electrode placement, the skin was prepared to optimize signal quality by trimming hair if necessary, gently abrading the skin surface, and cleaning the area with alcohol to reduce skin impedance. Surface electrodes (Ag/AgCl) were applied to the prepared sites. To enhance reproducibility, all electrode placements were performed by the same examiner following a consistent and standardized procedure. These electrode placement methods are considered to provide acceptable reliability in surface EMG measurements [28].

Surface EMG signals were acquired at a sampling rate of 1500 Hz using Myo-Research Master Edition 1.06 XP software (Noraxon U.S.A., Inc., Scottsdale, AZ, USA.). The raw signals were filtered using a 20–400 Hz band-pass filter in combination with a 60 Hz notch filter to reduce noise, as these filtering parameters reflect commonly adopted surface EMG processing practices aimed at minimizing motion artifacts and power-line interference while maintaining physiologically meaningful signal components. The processed signals were subsequently transformed into root mean square (RMS) values and normalized to each participant’s maximal voluntary isometric contraction (%MVIC).

MVIC was conducted based on Kendall’s manual muscle testing procedures [25]. To ensure measurement consistency, all MVIC assessments were conducted by a single experienced examiner under standardized conditions. Specifically, GM activity was assessed during hip abduction in a side-lying position, QL activity during pelvic hiking in the supine position with MR, and IO activity during a resisted trunk rotation maneuver in the supine position. Each contraction was maintained for 5 s, and the average value of the central 3 s interval was obtained after excluding the first and last seconds. To minimize fatigue, a 1 min rest was provided between trials. Measurements were repeated three times for each muscle, and the mean of the three trials was analyzed. The intra-session reliability of the three trials was assessed using the intraclass correlation coefficient (ICC(3,1)), showing high to excellent reliability (GM: 0.95, IO: 0.91, QL: 0.88). The ratio of GM to QL muscle activation was determined by comparing their mean EMG values. To ensure measurement consistency, all MVIC assessments were conducted by a single experienced examiner under standardized conditions.

### 2.3. Assessment of Lateral Pelvic Tilt Angle

LPT angle was evaluated during the SHA with a motion tracking system (ReLive, Gimhae, Republic of Korea), which utilizes inertial measurement unit (IMU) sensors to capture movement. To ensure temporal alignment of the data, LPT measurements were obtained simultaneously with EMG recordings. The sensor unit was secured over the mid-sacral region using double-sided adhesive tape, following previously established procedures. Throughout the movement, data were continuously acquired and wirelessly transmitted to a tablet device via Bluetooth. For analysis, the maximum LPT angle recorded in each trial was selected. This measurement approach is consistent with previous studies that utilized the same system to assess lumbopelvic kinematics [20]. To enhance measurement reliability and accuracy, sensor placement was standardized, all measurements were conducted by a single examiner under consistent conditions, and repeated trials were performed with the mean value used for analysis.

### 2.4. Experimental Protocol

Prior to data collection, all participants received standardized instruction on performing SHA and the ADIM. A familiarization session of approximately 10 min was conducted so that participants were able to correctly execute SHA with ADIM maintained. During ADIM training, a pressure biofeedback unit (PBU; Chattanooga, TN, USA) served to enhance activation of the abdominal musculature with participants positioned prone. Positioned beneath the abdominal region near the lumbar spine, the PBU was set to an initial pressure of 70 mmHg. Participants were subsequently instructed to gently contract the abdominal wall during expiration, aiming to maintain or modestly reduce the pressure within a range of 5–10 mmHg.

After confirming that participants could perform the maneuver consistently, they completed SHA across three conditions, namely ADIM-only, MR-only, and ADIM with MR. To reduce potential fatigue and carry-over effects, a 5 min rest interval was provided between each condition [13,16], and the sequence of conditions was randomly assigned. Each trial was performed using the dominant leg, which was identified based on the participant’s self-reported preferred limb.

Under the ADIM-only condition, participants were positioned in a straight side-lying posture, with the trunk and lower limbs maintained in a neutral alignment. The test limb was maintained in neutral alignment, and hip abduction was performed to a predetermined target angle of 30° using a goniometer. Participants were instructed to perform ADIM prior to initiating movement and to maintain it throughout the task. Once the target position was reached, an isometric contraction was held for 5 s. During testing, ADIM execution was monitored by the examiner through visual observation of abdominal movement and verbal confirmation of the drawing-in maneuver. To avoid increasing task complexity, a pressure biofeedback unit was not used during the test trials. Instead, trials were performed only after participants demonstrated correct execution, and any trial showing compensatory movement or loss of the drawing-in position was repeated. All tasks were performed three times with a 30 s rest period between trials. EMG signals were acquired during 5 s isometric contractions, and the average of the central 3 s interval was analyzed, with the initial and final seconds removed. The peak LPT angle recorded during each trial was used in statistical analysis.

Under the MR-only condition, SHA was performed without the use of ADIM. Manual resistance was applied at the distal lateral aspect of the thigh in the direction of hip abduction to induce isometric contraction at a 30° hip abduction position. The magnitude of resistance was individually adjusted according to each participant’s strength level to elicit maximal isometric contraction and maintain a stable position without visible movement [20]. All MR was applied by the first author, who has more than 16 years of clinical experience, to ensure consistency. Therefore, the magnitude of MR differed across participants and was not objectively quantified. All other procedures were identical to those used in the ADIM-only condition.

Under the ADIM with MR condition, participants performed SHA while maintaining ADIM, which was applied in the same manner as in the ADIM-only condition, and MR was applied using the same approach as in the MR-only condition. Other aspects of the protocol were kept consistent with those described previously.

### 2.5. Statistical Analysis

All statistical analyses were performed using PASW Statistics 18 (SPSS Inc., Chicago, IL, USA). Data normality was assessed using the Shapiro–Wilk test. Differences in EMG amplitudes of the GM, QL, and IO, as well as the GM/QL ratio and LPT angle across the three conditions, were analyzed using a one-way repeated-measures ANOVA. When the assumption of sphericity was violated, the Greenhouse–Geisser correction was applied. Post hoc comparisons were conducted with Bonferroni adjustment. Effect sizes were calculated using partial eta squared (*η_p_*^2^) for ANOVA and Cohen’s d (*d_z_*) for pairwise comparisons. Statistical significance was set at *p* < 0.05.

## 3. Results

Significant differences among the three conditions were observed in the muscle activity of the GM, QL, IO, and the GM/QL ratio (*p* < 0.05) (Table 2). The Shapiro–Wilk test indicated that most variables met the normality assumption (*p* > 0.05), Although one variable showed a minor deviation from normality, parametric analysis was retained because repeated-measures ANOVA is generally robust to modest deviations from normality in within-subject designs, particularly with a sample size of 22.

Pairwise comparisons demonstrated condition-specific differences, with effect sizes calculated using paired-samples Cohen’s d (*d_z_*). GM activity was higher under MR-only (*d_z_* = 2.52) and ADIM with MR (*d_z_* = 2.26) compared to ADIM-only (*p* < 0.001). For QL, MR-only produced greater activation than both ADIM-only (*d_z_* = 2.77) and ADIM with MR (*d_z_* = 0.69), while ADIM with MR also exceeded ADIM-only (*d_z_* = 2.25) (*p* < 0.05). IO activity peaked in the ADIM with MR condition (*d_z_* = 2.14 vs. ADIM-only; *d_z_* = 0.81 vs. MR-only), with MR-only still exceeding ADIM-only (*d_z_* = 1.41) (*p* < 0.05). The GM/QL ratio increased under ADIM-only (*d_z_* = 0.75) and ADIM with MR (*d_z_* = 0.58) relative to MR-only (*p* < 0.05).

**Figure 1:**
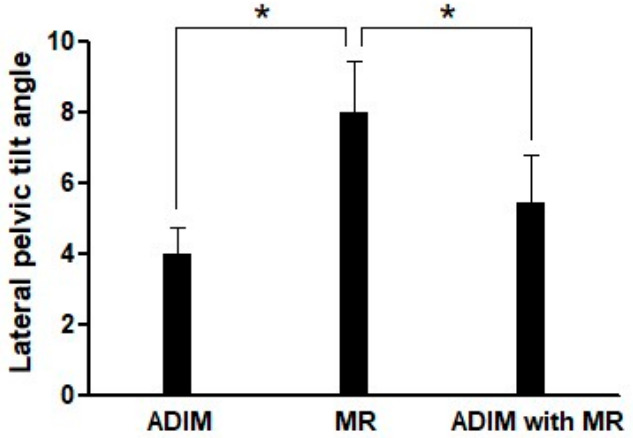
Lateral pelvic tilt angle during side-lying hip abduction among three conditions. ADIM, abdominal draw-in maneuver; MR, manual resistance; * *p* < 0.05.

The LPT angle differed significantly among the three conditions (*F* = 73.12, *p* < 0.001, *η_p_*^2^ = 0.777) (Figure 1). The MR-only condition showed the highest LPT angle (8.03 ± 1.43), whereas lower angles were observed in the ADIM with MR (5.47 ± 1.34, *d_z_* = 1.38) and ADIM-only (4.01 ± 0.76, *d_z_* = 2.77) conditions (*p* < 0.001).

## 4. Discussion

Applying the ADIM during SHA has been shown to increase the activation of the transversus abdominis and IO, thereby contributing to enhanced lumbopelvic stability [13,16]. This improved stability can attenuate compensatory activity of the QL and limit excessive LPT, facilitating more selective activation of the GM [14,17,18]. Furthermore, MR is widely recognized as an effective strategy for enhancing target muscle activation through the application of controlled resistance vectors and individualized loading conditions [15,20,26]. Therefore, the present study aimed to examine and compare how ADIM, MR, and their simultaneous application during SHA influence the activation of the GM, QL, and IO, as well as the GM/QL activity ratio and LPT angle. The GM/QL ratio was used as an index to reflect the relative balance between the GM and QL, where a higher ratio indicates more preferential activation of the GM relative to the QL [17,19].

In the present study, the ADIM with MR condition increased muscle activation in all measured muscles relative to the ADIM-only condition (*p* < 0.05), whereas no significant differences were observed in the GM/QL ratio or LPT angle. Previous studies on SHA have primarily focused on reducing QL activity while increasing GM activation [13,16,19]. However, in the current study, QL activity also increased under the ADIM with MR condition (*p* < 0.05). Considering that no meaningful differences were identified in the GM/QL ratio and LPT angle between the two conditions, it is limited to interpret the increased QL activity as contributing to greater LPT. Furthermore, as GM activity also increased significantly, these findings might not be solely attributed to compensatory activation of the QL. Supporting this interpretation, Oshikawa et al. [29] reported that the QL, together with the transversus abdominis, plays an important role in controlling the center of mass and stabilizing the lumbar spine in the frontal plane. Taken together, the increased QL activity observed under the ADIM with MR condition could be interpreted as a greater overall muscular demand required to resist isometric loading and maintain stable SHA performance, rather than purely compensatory activation. Alternatively, it may be associated with increased trunk stabilization demands due to external resistance, potentially reflecting a global co-contraction pattern. Therefore, the interpretation of increased QL activity should be approached with caution.

A comparison between the MR-only condition and the ADIM with MR condition revealed that QL activity was significantly lower, whereas IO activity and the GM/QL ratio were significantly higher, and LPT angle was significantly reduced under the ADIM with MR condition (*p* < 0.05). Although no significant difference was observed in GM activity between the two conditions, the increased GM/QL ratio together with the reduction in LPT angle (*p* < 0.05) may indicate a more favorable muscle recruitment pattern between the GM and QL, potentially reflecting improved lumbopelvic control. These findings are consistent with the previous literature, which suggests that optimizing muscle recruitment patterns and pelvic alignment can reduce the risk of compensatory strategies during SHA [13,14,15,16]. However, this interpretation should be made with caution, as functional outcomes were not directly assessed in the present study.

The lack of a significant difference in GM activity may be attributed to the inherent nature of the SHA exercise, which requires high levels of GM activation regardless of ADIM application. Furthermore, the combined application of ADIM and MR may facilitate co-contraction of the IO and transversus abdominis, which may contribute to enhanced lumbopelvic control [20]. Although transversus abdominis activity was not directly measured in this study, previous studies have used IO activity as a surrogate marker of its activation [30,31]. However, it is important to acknowledge the distinct differences between these two muscles. The IO is a more superficial muscle primarily involved in trunk rotation. In contrast, the transversus abdominis is the deepest layer responsible for maintaining postural stability and providing lumbopelvic support. Therefore, IO activity has an inherent limitation in that it does not directly represent the isolated function of the transversus abdominis. Nevertheless, as the IO contributes to lumbopelvic stability, the increased IO activity observed under the ADIM with MR condition may be associated with stabilization.

The results of the present study showed that both the MR-only and ADIM with MR conditions led to significant increases in muscle activation across all measured muscles relative to the ADIM-only condition (*p* < 0.05), with noticeable differences also observed in absolute values. This finding may be explained by the increased external load, which requires greater force production and motor unit recruitment to maintain the target position, along with increased demands for intermuscular coordination and co-contraction to stabilize the lumbopelvic region. Previous studies have reported that resistance training induces neuromuscular adaptations such as improved coordination and increased motor unit recruitment [32,33]. Consistent with these findings, the increased muscle activation observed in the present study may reflect an acute neuromuscular response to external resistance.

Kim and Shin [20] reported that the simultaneous application of ADIM and MR during prone hip extension increased IO muscle activity and reduced anterior pelvic tilt, suggesting its usefulness for improving lumbopelvic stability. In the present study, the ADIM with MR condition also resulted in significantly greater IO muscle activity compared to the other conditions and significantly reduced LPT angle compared to the MR-only condition (*p* < 0.05). In contrast, Dafkou et al. [34] applied ADIM with external loads of 10 kg and 20 kg using a barbell during the supine bridge exercise and reported that such external resistance did not significantly enhance abdominal muscle activation. The discrepancy between these findings may be attributed to differences in the type and application of resistance. External resistance using objects provides a constant load in the direction of gravity, whereas MR offers adaptive resistance that can be adjusted dynamically based on individual performance and applied at an optimal angle [20,26]. These characteristics may facilitate more effective preferential activation of target muscles, thereby enhancing motor control strategies.

Several limitations of the present study should be acknowledged. The sample size was relatively small (*n* = 22), which may limit the generalizability of the findings. The sample consisted of healthy males, which may limit the applicability of the findings to female populations, different age groups, and clinical populations, including individuals with Trendelenburg gait. Additionally, although individuals with professional or regular resistance training backgrounds were excluded, participants’ daily physical activity levels were not quantitatively assessed. Therefore, the influence of individual differences in baseline neuromuscular efficiency cannot be ruled out. Although MR effectively induced muscle activation, its mechanical intensity was not quantitatively measured in units such as Newtons, limiting the ability to establish a standardized resistance protocol. Moreover, due to the nature of the intervention, blinding of the examiner and participants was not feasible, which may have introduced potential bias. In addition, due to the cross-sectional design of this study, only acute neuromuscular responses during SHA were examined. Therefore, the long-term cumulative effects of combined ADIM and MR training remain unclear. Furthermore, the use of surface EMG to assess QL activity may be limited due to muscle depth and potential crosstalk from adjacent muscles. Moreover, only the peak LPT value was analyzed, and temporal characteristics and movement variability during SHA were not assessed. Finally, although changes in IO and QL activity and LPT were observed, we did not directly assess the transversus abdominis or deep hip rotator muscles, which also play important roles in lumbopelvic control.

## 5. Conclusions

In conclusion, the simultaneous application of ADIM with MR during SHA significantly increased IO activation and the GM/QL ratio, while reducing LPT. These findings suggest a more favorable muscle activation pattern and potentially improved lumbopelvic control. Therefore, the combined application of ADIM with MR may serve as a potential strategy for optimizing motor control and muscle activation during SHA. However, as the present study examined only acute neuromuscular responses, further longitudinal studies are needed to determine the long-term effects and potential clinical relevance of this approach.

## Figures and Tables

**Table 1:** Participant characteristics (*n* = 22).

Variables	Mean ± SD
Age (year)	23.58 ± 1.96
Height (cm)	173.85 ± 5.21
Weight (kg)	66.42 ± 6.14
BMI	21.98 ± 1.63

SD, standard deviation.

**Table 2:** Muscle activity during side-lying hip abduction across three conditions.

Muscle Activity (%MVIC)	ADIM (95% CI)	MR (95% CI)	ADIM with MR (95% CI)	*F*	*η_p_* ^2^	*p*
Gluteus medius	45.93 ± 10.68 ^a,b^ (41.19–50.66)	78.41 ± 8.28 (74.74–82.09)	80.04 ± 8.33 (76.34–83.73)	90.52	0.812	<0.001 *
Quadratus lumborum	27.90 ± 7.83 ^a,b^ (24.43–31.37)	61.89 ± 9.61 ^c^ (57.63–66.15)	52.38 ± 8.71 (48.52–56.24)	88.73	0.809	<0.001 *
Internal oblique	34.93 ± 8.69 ^a,b^ (31.08–38.78)	49.30 ± 8.52 ^c^ (45.52–53.07)	58.45 ± 8.58 (54.65–62.26)	52.65	0.715	<0.001 *
Gluteus medius/ Quadratus lumborum ratio	1.76 ± 0.61 ^a^ (3.68–4.35)	1.30 ± 0.27 ^c^ (7.39–8.66)	1.57 ± 0.35 (4.88–6.06)	6.30	0.231	0.007 *

Data are expressed as mean ± standard deviation. MVIC, maximal voluntary isometric contraction; ADIM, abdominal draw-in maneuver; MR, manual resistance; * *p* < 0.05; ^a^ significant difference between ADIM-only and MR-only conditions; ^b^ significant difference between ADIM-only and ADIM with MR conditions; ^c^ significant difference between MR-only and ADIM with MR conditions.

## Data Availability

Data supporting the reported results are not publicly available due to privacy/ethical restrictions.

## Abbreviations

The following abbreviations are used in this manuscript:

SHA	Side-Lying Hip Abduction
GM	Gluteus Medius
QL	Quadratus Lumborum
IO	Internal Oblique
LPT	Lateral Pelvic Tilt
ADIM	Abdominal Draw-In Maneuver
MR	Manual Resistance
